# Prevention of allergic airway hyperresponsiveness and remodeling in mice by *Astragaliradix* Antiasthmatic decoction

**DOI:** 10.1186/1472-6882-13-369

**Published:** 2013-12-25

**Authors:** Su Xu, Bao-Ping Tian, Lan-Hong Zhang, Wen Hua, Li-Xia Xia, Zhi-Hua Chen, Wen Li, Hua-Hao Shen

**Affiliations:** 1 Department of Respiratory and Critical Care Medicine, Second Affiliated Hospital of Zhejiang University School of Medicine, Medicine, 88 Jiefang Rd., Hangzhou 310009, China; 2 State Key Laboratory of Respiratory Diseases, Guangzhou 510120, China

**Keywords:** *Astragalus* Antiasthmatic Decoction, Airway hyperresponsiveness, Airway remodeling

## Abstract

**Background:**

*Astragali radix* Antiasthmatic Decoction (AAD), a traditional Chinese medication, is found effective in treating allergic diseases and chronic cough. The purpose of this study is to determine whether this medication could suppress allergen-induced airway hyperresponsiveness (AHR) and remodeling in mice, and its possible mechanisms.

**Methods:**

A mouse model of chronic asthma was used to investigate the effects of AAD on the airway lesions. Mice were sensitized and challenged with ovalbumin (OVA), and the extent of AHR and airway remodeling were characterized. Cells and cytokines in the bronchoalveolar lavage fluid (BALF) were examined.

**Results:**

AAD treatment effectively decreased OVA-induced AHR, eosinophilic airway inflammation, and collagen deposition around the airway. It significantly reduced the levels of IL-13 and TGF-β1, but exerted inconsiderable effect on INF-γ and IL-10.

**Conclusions:**

AAD greatly improves the symptoms of allergic airway remodeling probably through inhibition of Th2 cytokines and TGF-β1.

## Background

Asthma is a chronic airway inflammatory disease, which is characterized by airway hyperresponsiveness (AHR), chronic pulmonary inflammation with infiltration of eosinophils in lung, and a predominant T helper (Th) 2 immune response. The persistent Th2 response eventually leads to airway remodeling, characterized by increased goblet cell hyperplasia and mucus hyper-production, smooth muscle hypertrophy, excessive collagen deposition, and increased angiogenesis, and consequently results in persistent AHR and irreversible airway obstruction with pulmonary function depression [1],[2].

The Th2 cells secrete several proinflammatory cytokines, such as IL-4, IL-5 and IL-13, among which IL-5 promotes differentiation and migration of eosinophils, while IL-4 and IL-13 play pivotal roles in goblet cell hyperplasia, the development of AHR, and airway remodeling. On the contrary, antigen-induced Th1 responses and associated IFN-γ production can down-regulate Th2 responses thereby inhibiting allergen-induced airway inflammation [3],[4]. Accumulating evidence has suggested that the regulatory T cells (Treg) and their released cytokines such as IL-10 are also capable of attenuating the Th2 response and therefore play a protective role in allergic asthma [5],[6].

*Astragali radix,* the dried roots of *Astragalus membranaceus* (Fisch.) Bge., is a traditional Chinese herb which has been widely used in a variety of medications for more than 2000 years in China. This herb is used as a potent tonic for increasing energy levels and stimulating the immune system by the traditional Chinese doctors, and the possible molecular mechanisms mediating its therapeutic effects have also been investigated [7],[8]. We have also observed that *Astragali radix* prevented AHR in mice with an inhibition of Th2 response [9], and that *Astragali*-*Cordyceps* Mixtura ameliorated asthmatic airway remodeling through modulation of the TGF-β1/Smad signaling cascade [10].

However, most of the traditional Chinese medications or patent drugs generally comprise a number of herbs, keeping the drug as dialectical, conciliatory, and balanceable. *Astragali radix* Antiasthmatic decoction (AAD) is such a traditional Chinese drug formula comprising 9 kinds of Chinese herbs (detailed in Methods below). This medication is designed to harmony the functions of lung, kidney and spleen, which has been demonstrated to be as effective as budesonide in treating allergic asthma and chronic cough [11]. In the present study, we explored the role of these herbal formulae in allergic mouse AHR and airway remodeling, and investigated its possible underlying molecular mechanisms.

## Methods

### Preparation of AAD

AAD, comprising 9 traditional Chinese herbs, *Astragali radix,* the drided roots of *Astragalus membranaceus* (Fisch.) Bge. Huangqi, 15 g; *Codonopsis radix*, the dried roots of *Codonopsis pilosula* (Franch.) Nannf., Dangshen, 10 g; *Atractylodis macrocephalae rhizoma*, the fried and dried rhizome of *Atractylodes macrocephala* Koidz., Chao Baizu, 15 g; *Psoraleae fructus*, the dried fruits of *Psoralea corylifolia* L., Buguzhi, 10 g; *Cuscutae semen*, the dried seeds of *Cuscuta chinensis* Lam., Tusizi, 10 g; *Schisandrae chinensis fructus*, the dried fruits of *Schisandra chinensis* (Turcz.) Baill., Wuweizi, 6 g; *Citri reticulatae pericarpium*, the dried mature peels of *Citrus reticulata* Blanco, Chenpi, 6 g; *Pinelliae rhizome*, the dried tuber of *Pinellia ternate* (Thunb.) Breit., Banxia, 10 g; and *Saposhnikoviae radix*, the dried roots of *Saposhnikovia divaricata* (Turcz.) Schischk., Fangfeng, 10 g; was a traditional Chinese decoction widely used to treat asthma and chronic cough for more than one thousand years. The criteria for the quality of the herbs used were in accordance with the 2010 Chinese pharmacopoeia. The decoction was prepared according to a standardized procedure. Briefly, the medicinal herbs mixture (92 g) was homogenized to a fine powder and then extracted twice in a reflux condenser for 2 h with 75% ethanol in a heated water-bath and the resulting aqueous extracts spray dried to obtain a powder. Subsequently, the pooled extract was filtered to remove debris, and the ethanol was removed by rotary evaporation under reduced pressure. The concentrated extract was then dried by lyophilization to obtain the AAD extract. The extract powder was used for animal experiments by dissolution in pure water at the following different concentrations of suspension (0.375 kg crude drugs/L and 1.50 kg crude drugs/L).

#### Mouse model of airway remodeling and AAD treatment

Eight to ten weeks old BALB/c mice were purchased from the Experimental Animal Center of Zhejiang University and housed in specific pathogen-free facility. All protocols in this study were approved by the Ethical Committee for Animal Studies at Zhejiang University, China (Register Number: 2011030101). Mice (8–10 mice/group) were immunized on days 0 and 14 via intraperitoneal injection with 20 µg of OVA (Sigma-Aldrich, St Louis, Missouri) emulsified in 100 µL Imject Alum (Pierce, Rockford, Illinois). Groups of mice that had been sensitized with OVA were challenged with aerosolized OVA (1% OVA in saline for 40 minutes) for 18 days consecutively. Two hours before challenging, mice were treated with AAD in half (7.5 g crude drugs/kg/d,) or equal (15.0 g crude drugs/kg/d, each ml of solution contained 15.0 g crude drug) doses used in human (L-OVA and H-OVA, respectively) or physiological saline (OVA group) by intragastric administration once a day till the last challenge.

#### Assessment of airway hyperresponsiveness

Twenty-four hours after the final OVA challenge, airway hyperresponsiveness (AHR) to inhaled methacholine (Meth, Sigma-Aldrich) was measured as described previously [12]. Mice were anesthetized, and the tracheas were cannulated to an inline aerosol administrator and ventilator, which were attached to a preamplifier and computer (Buxco, Wilmington, North Carolina). Mice were nebulized with PBS followed by increasing doses of Meth (6, 12, 25 mg/mL). At each dose, lung resistance was calculated.

#### Assessment of airway inflammation

Collection of BALF was performed 48 h after the final OVA challenge, as previously described [12]. In brief, inflammatory cells were obtained by cannulation of the trachea and lavage of the airway lumen with 0.8 ml PBS. Cytospin slides were prepared by Wright-Giemsa staining. Total cell and eosinophil counts were counted under the microscope in a blinded method.

#### Lung histology and determination of collagen deposition

The left lobes of lungs were fixed in 10% buffered formalin. After paraffin-embedding, their tissue sections were prepared (3 µm). Lung sections were stained with hematoxylin/eosin (H&E) or Masson trichrome. The HE staining sections were semi-quantitated (score: 0–4) with Olympus microscope (10 × 20 magnification) for the inflammatory situation. Collagen area on the basal membrane of airway was analyzed by Leica-Qwin image-processing system (Leica Imaging Systems, Bensheim, Germany). The result was expressed as collagen staining area of per micrometer length of basement membrane of bronchioles. At least 10 bronchioles with 150 to 250 µm of internal diameter were counted on each slide. All slides were examined in a random blinded fashion by 2 independent investigators.

### Analysis of cytokines

The concentrations of IFN-γ, IL-10, IL-13, and TGF-β in BALF were measured using a commercially available ELISA kit (R&D systems, Minneapolis, MN) according to the manufacturer’s protocols.

#### Statistical analysis

Data are expressed as mean ± SE. Differences of normally distributed data between the groups were analyzed by one-way analysis of variance (one-way ANOVA) or by an unpaired Student’s t test using SPSS 13.0 software. A value of P ≤ 0.05 was considered statistically significant.

## Results

### AAD prevented AHR in chronic OVA-challenged mice

**Figure 1:**
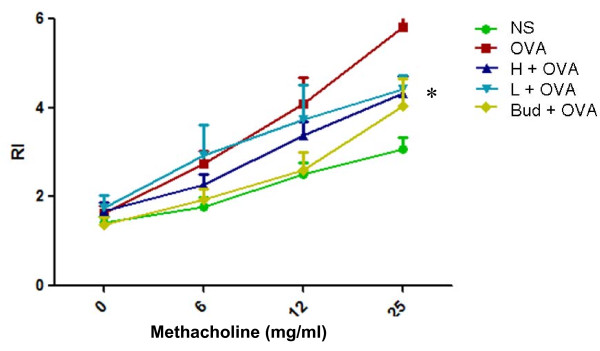
**AAD prevented AHR in chronic OVA-challenged mice.** Airway hyperresponsiveness (AHR) of mice was determined in the means of response to methacholine on 24 h after the last OVA (or NS) challenge by using the Buxco system as described in the Methods section. Data are presented as means ± SE for 8–10 mice in each group of three independent experiments. H + OVA: OVA challenged and treated with high dose AAD, L + OVA: OVA challenged and treated with low dose AAD. *P < 0.05 compared with the data of OVA group.

To determine whether AAD treatment improves pulmonary function of allergic mice after chronic challenge with OVA, an invasive method was used to determine the levels of AHR in response to Meth. Exposure of mice to chronic OVA challenges induced a significant increase in AHR compared with saline controls (Figure 1). However, AAD treatment at either dose significantly decreased AHR compared with OVA group in response to Meth at 25 mg/ml (Figure 1), and there were no significant differences between these two groups treated with different doses of AAD.

#### AAD reduced total leukocytes and eosinophils in the BALF of allergic mice

**Figure 2:**
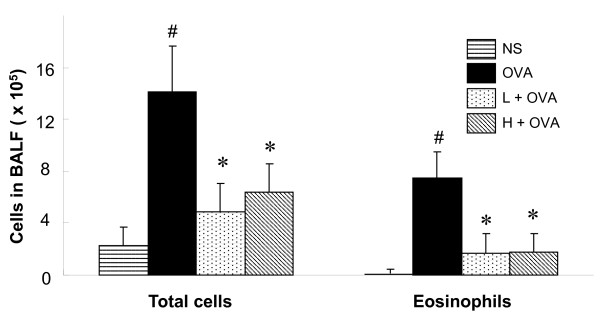
**AAD reduced total leukocytes and eosinophils in the BALF of allergic mice.** The bronchoalveolar lavage fluid (BALF) of mice were harvested at 48 h after the final OVA (or NS) challenge. Subsequently, total leukocytes cell counts (Left) were carried out using a Neubauer chamber. Smears of BALF cells were prepared and eosinophil counts (Right) were performed in light microscopy according to standard morphologic criteria by the smears following Wrights-Giemsa staining, at least 400 cells were counted per slide. The values represent the mean ± SE for 8–10 mice in each group of three independent experiments. H + OVA: OVA challenged and treated with high dose AAD, L + OVA: OVA challenged and treated with low dose AAD. ^#^p < 0.05 compared with NS. *P < 0.05 compared with OVA group.

Exposure of mice to chronic OVA challenges induced a significant increase in the numbers of total cells and eosinophils in BALF compared with their controls (Figure 2). However, AAD treatment significantly decreased levels of total cells and eosinophils in BALF (Figure 2). Other cells such as neutrophils, monocytes, and lymphocytes were only slightly affected by AAD treatment (data not shown). In agreement with the results of AHR in Figure 1, the two doses of AAD (7.5 or 15.0 g/kg/d) again exhibited similar protective effect on the OVA-induced airway inflammation.

#### AAD ameliorated OVA-induced inflammatory infiltration in lung tissue of mice

**Figure 3:**
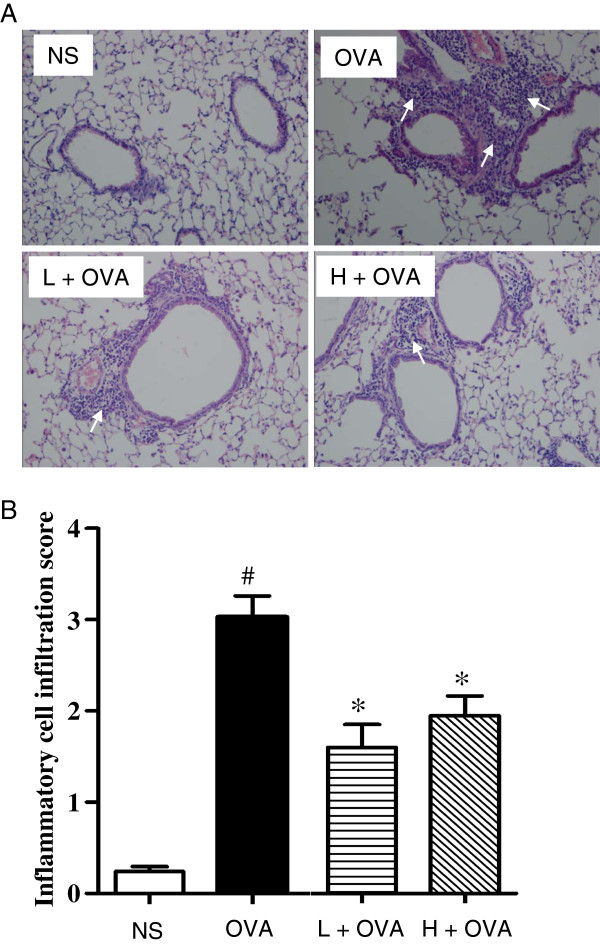
**AAD ameliorated Ova-induced inflammatory infiltration in lung tissue of mice.** Histologic analysis of the lung sections were performed with HE to visualize inflammatory cell recruitment, representative photomicrographs of lung inflammation expression are shown **(A)**. White arrows indicate areas with clear infiltrated inflammatory cells. With semi-quantitated (score: 0–4) under Olympus microscope (10 × 20 magnification), the level of inflammatory cell infiltration **(B)** was assessed by two observers independently. Data are presented as means ± SE for 8–10 mice per group. H + OVA: OVA challenged and treated with high dose AAD, L + OVA: OVA challenged and treated with low dose AAD. ^#^p < 0.05 compared with NS. *P < 0.05 compared with OVA group.

To further explore the effect of AAD on prevention of allergic airway inflammation, we also examined the pulmonary pathology stained with H&E in mice of all groups. Chronic exposure to OVA resulted in the development of a characteristic peribronchial and perivascular airway tissue infiltration compared with mice treated with saline (Figure 3A). Again, the AAD treatment at either dose significantly reduced the inflammatory cell infiltration (Figure 3A). A semi-quantitative analysis also further revealed that the inflammation scores of peribronchial and perivascular regions were significantly decreased in the AAD treated groups relative to OVA group (Figure 3B).

#### AAD decreased the collagen deposition in the model of chronic asthma

**Figure 4:**
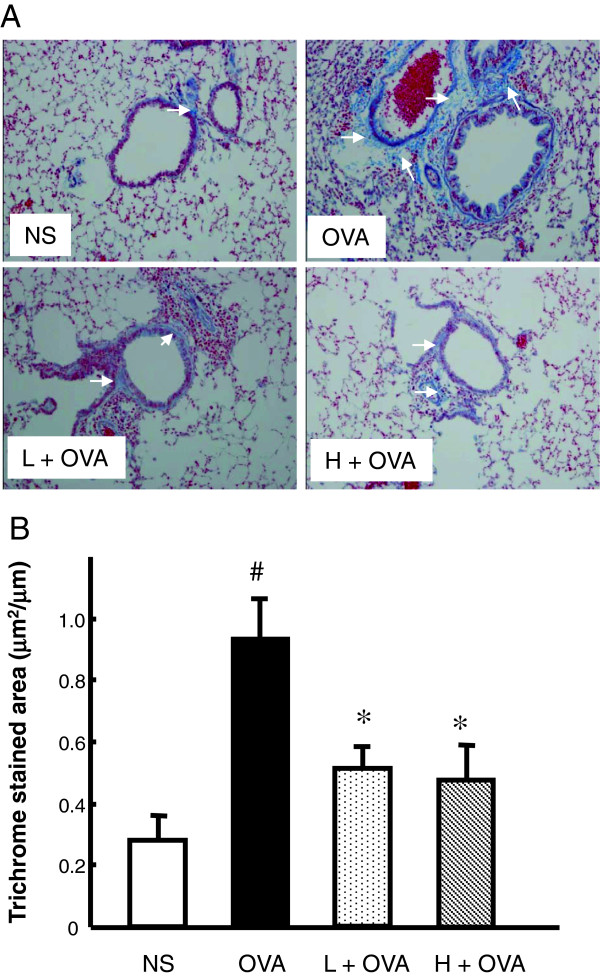
**AAD decreased the collagen deposition in the model of chronic asthma.** Lung sections were stained with Masson trichrome to detect collagen deposition around airways. Representative photomicrographs of collagen deposition in different group are shown in **(A)**. White arrows indicate areas with clear collagen deposition. Quantitatively, collagen area on the basal membrane of airway was analyzed and the result was expressed as collagen staining area per micrometer length of basement membrane of bronchioles **(B)**. Data are presented as means ± SE for 8–10 mice per group. H + OVA: OVA challenged and treated with high dose AAD, L + OVA: OVA challenged and treated with low dose AAD. ^#^p < 0.05 compared with NS. *P < 0.05 compared with OVA group.

To examine the airway remodeling induced by chronic OVA exposure, we detected the collagen deposition using Masson trichrome staining. As shown in Figure 4, there was a little collagen deposition around airway walls and blood vessels in the normal mice, while it was markedly increased in the chronic asthma model (Figure 4). AAD treatment at either dose effectively reduced the OVA-induced collagen deposition (Figure 4).

#### AAD modulated the levels of various cytokines in BALF of mice

**Figure 5:**
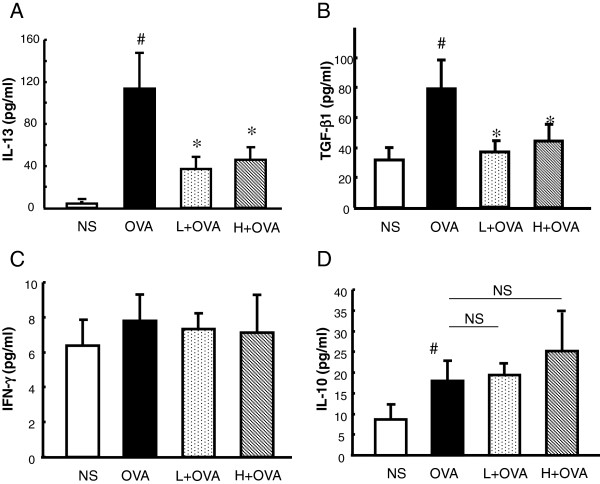
**AAD modulated the levels of cytokines in BALF of mice.** The BALF was harvested at 48 h after the final OVA (or NS) challenge. Levels of IL-13 **(A)**, TGF-β **(B)**, IFN-γ **(C)**, and IL-10 **(D)** in the supernatant of BALF were evaluated by ELISA. Data are presented as means ± SE for 8–10 mice per group. H + OVA: OVA challenged and treated with high dose AAD, L + OVA: OVA challenged and treated with low dose AAD. ^#^p < 0.05 compared with NS. *P < 0.05 compared with OVA group. NS means not significant.

To evaluate the effect of AAD on the pulmonary immune response in allergic mice, the concentrations of IL-13, IL-10, IFN-γ and TGF-β1 in BALF were measured. Chronic OVA challenges induced marked increases of IL-13 and TGF-β1, both of which were significantly attenuated by AAD treatment (Figure 5A&B). The levels of IFN-γ were comparable among all groups, affected by neither OVA nor AAD treatment (Figure 5C). The levels of IL-10 were significantly increased in OVA challenged mice (Figure 5D). AAD treatment only slightly increased the expression of IL-10 compared to the OVA group, but it did not reach statistic significance (Figure 5D).

## Discussion

Our present study clearly demonstrated that AAD effectively prevented AHR, inflammation, and airway remodeling in a mouse model of chronic asthma. Although AAD exerted similar levels of inhibitory effect compared with the results that *Astragali radix* could significantly attenuate AHR [9], and that Astragali-Cordyceps Mixtura could ameliorate asthmatic airway remodeling [10], this patent drug should apparently have its advantages in vivo, as it comprise of a number of herbs and could harmony the functions of lung, kidney and spleen. In general, the multiple mixture medication should be more dialectical, more conciliatory, and more balanceable than a single herb. Moreover, there were not any appreciable differences between the protective effects of the two doses of AAD used throughout the study, suggesting that this patent drug might be used at a lower dose for treating allergic patients to reduce its possible side effects.

Asthma has long been thought to be a Th2-driven disease, and the Th2 related cytokines are known to play important roles in the pathogenesis of asthma. Among these Th2 cytokines, IL-13 has been demonstrated to be a critical mediator in the mucus obstruction phenotype evidenced in the mouse model of allergic asthma [13]. Pulmonary aspiration of IL-13 alone could cause airway epithelial thickening and goblet cell hyperplasia [14],[15]. Transgenic overexpression of IL-13 in the airways markedly induced airway inflammation and mucus hyperplasia [16]. In our study, we observed that AAD significantly inhibited the expression of IL-13, which was in agreement with our previous results that *Astragali radix* could inhibit Th2 cytokines. Apparently, this IL-13 inhibition should contribute to the overall protective effects of AAD in allergic airway inflammation and remodeling.

TGF-β1 has been known to play a pivotal role in the airway remodeling. It is one member of the family of structurally related growth factors expressed in a variety of lung cells such as epithelium, endothelium, alveolar macrophage, fiber archeocyte, and smooth muscle cells. It has been shown to exert important effects in airway fibrosis and collagen deposition in asthmatic airway remodeling [17]-[19]. We have previously observed that Astragali-Cordyceps Mixtura significantly down-regulated TGF-β1 and its related signaling cascades. In line with this, our present study also demonstrated that AAD effectively attenuate TGF-β1 expression induced by chronic allergen exposure. Thus, the inhibition of TGF-β1 by AAD could eventually prevent subsequent collagen deposition and airway remodeling.

It might be noteworthy that the levels of IFN-γ were not affected by the AAD treatment in Balb/c mice in our current study (Figure 5C), whereas we have observed that *Astragali radix* alone induced a marked elevation of IFN-γ in C57BL/6 mice [9]. One plausible explanation is that *Astragali radix* could significantly induce IFN-γ in the Th1-prone C57BL/6 mice while it failed to affect the Th1-IFN-γ pathway in the Th2-prone Balb/c mice due to the genetic differences of the mouse strains. It is also likely that *Astragali radix* alone and the AAD mixtura might exert differential effects on the Th1-IFN-γ signaling. In addition, we also examined the levels of IL-10 (Figure 5D) which may reflect the levels of regulatory T cells (Treg) in the local airways, and found that AAD exerted no considerable effect on the expression of IL-10. Collectively, these data suggested that AAD prevented the Th2 response and airway remodeling probably not through induction of Th1 and Treg, while the detailed mechanisms require further investigations.

Besides *Astragali radix*, other ingredients of AAD have also been shown to exert certain protective effect in respiratory diseases including allergic inflammation. For example, *Atractylodis macrocephalae* rhizome has been reported to inhibit infections in the upper respiratory tract, and also to significantly decrease the immune responses elicited by antigen ovalbumin in mice [20]. *Pinelliae* rhizome and *Citrus reticulata* and their combinational prescription have deep inhibitory effects on airway inflammation in a murine model of asthma and it was mediated by suppression of Th2 cytokines (IL-4, IL-5, IL-13), IgE, eosinophil CCR3 expression in lung [21]. In addition, *Psoraleae* fructus is commonly used in Chinese to alleviate asthma [22]. Other herbs [23]-[29], *Codonopsis* radix, *Cuscutae semen*, *Schisandrae chinensis* fructus*. Citri reticulatae pericarpium*, and *Saposhnikoviae* radix*,* have also been shown to have effective anti-inflammatory activities and modulated immune responses, which may also exert a preventive effect in allergic airway inflammation. The anti-inflammatory property of these herbs might contribute, more or less, to the overall protective effect of AAD in allergic inflammation.

In summary, the current study demonstrated that a traditional Chinese medication AAD could effectively prevent airway inflammation and remodeling in allergic mice through down-regulation of Th2-related cytokines and TGF-β1, suggesting a high potential of this medication in clinical therapy of allergic airway diseases.

## Conclusions

In conclusion, the present study served to confirm the therapeutic effects of AAD in the inhibition of eosinophilic airway inflammatory in vivo. AAD treatment effectively decreased OVA-induced AHR, eosinophilic airway inflammation, and airway remodeling, which probably through decreased expression of IL-13 and TGF-β1.

## Abbreviations

AAD: Astragalus Antiasthmatic Decoction; AHR: Airway heperresponsiveness; OVA: Ovalbumin; H&E: Hematoxylin/eosin; CCR3: CC chemokine receptor 3; Mch: Methacholine; BALF: Bronchoalveolar lavage fluid; NS: No significant.

## Competing interests

The authors declare that they have no competing interests.

## Authors’ contributions

SHH, LW, and CZH contributed to the study design, analysis, and interpretation of data. SHH conceived the study. XS, TBP, ZLH, HW, and XLX performed the experiments. XS and TBP participated in statistical analysis. XS, TBP, and CZH drafted the manuscript. All authors approved the final manuscript.

## Pre-publication history

The pre-publication history for this paper can be accessed here:

http://www.biomedcentral.com/1472-6882/13/369/prepub
